# Infantile gastroesophageal reflux in a hospital setting

**DOI:** 10.1186/1471-2431-8-11

**Published:** 2008-03-27

**Authors:** Susan S Baker, Christine M Roach, Michael S Leonard, Robert D Baker

**Affiliations:** 1 Department of Pediatrics University at Buffalo, Women and Children's Hospital, 219 Bryant Street, Buffalo, NY 14222, USA

## Abstract

**Background:**

Gastroesophageal reflux is a common diagnosis in infants. Yet, there is no information on the demographics of those hospitalized with reflux. The aim of this study is to describe the demographics of children with gastroesophageal reflux discharged from the hospital during the first two years of life.

**Methods:**

Retrospective chart review of children aged 0–2 years discharged between January 1, 1995 and December 31, 1999 with a diagnosis of reflux documented in their hospital chart prior to 12 months of age.

**Results:**

Reflux was the seventh most common reason for hospitalization. About 50% of subjects with reflux had multiple hospitalizations. Of the 1,096 infants diagnosed with reflux about half were born prematurely. Reflux was the primary diagnosis for 21% of all infants; 10% of those born prematurely. The average length of stay for the subjects was longer than the hospital average. African Americans, 2.4% of the population, accounted for 29% of discharges. Caucasians, 86% of the population, were 66% of discharges. 21.8% of African Americans and 68.3% of Caucasians were diagnosed with reflux. 35% of mothers smoked, 27% worked and 48% had public insurance, compared to 22.2%, 57%, and 24% respectively of females in the general population.

**Conclusion:**

Reflux is a common discharge diagnosis. Children who have primary reflux have longer than average hospital stays. About half had multiple admissions. Mothers of children with reflux are more likely to be less educated, receive public insurance, smoke, and be unemployed than the general female population in Western New York. Although African American children were disproportionately hospitalized, they were less likely to be diagnosed with reflux.

## Background

Gastroesophageal reflux, defined by the North American Society for Pediatric Gastroenterology, Hepatology and Nutrition as the passage of gastric contents into the esophagus [1], is common in infants [2,3]. Yet, there is no information on the demographics of children who have reflux and require hospitalization. Most often the clinical diagnosis of reflux and the decision to employ therapeutic interventions is made by clinicians based on signs and symptoms presented by the patient. Therefore, we used the diagnosis that clinicians assigned to their hospitalized patients to identify patients for inclusion in the study. For this report, we defined primary reflux as reflux that was the primary reason for hospitalization and secondary reflux as reflux that was not the primary reason for hospitalization.

We hypothesized that reflux is an important cause for hospitalizations for children ≤ 2 years of age, that reflux frequently occurs concomitantly with other diseases and that infants born prematurely (< 37 weeks gestation) who were admitted to the hospital with reflux had different characteristics than those who were born at term (≥ 37 weeks gestation). We also hypothesized that demographic data might identify children diagnosed with reflux who are at increased risk for hospitalization. This study was designed to describe the incidence, evaluation, and treatment of children with the most severe reflux, those requiring admission to the hospital, during the first two years of life. This manuscript reports on the demographics of the study children. The data did not allow us to determine the extent reflux contributed to the child's illness, only that it was present. Because the study contained thousands of data points for each category, this report focuses only on the demographic characteristics of the patients. Laboratory, therapeutic, financial and outcomes of the study patients will be presented in subsequent manuscripts.

## Methods

### Chart Review

This study was approved by the Children's and Youth Institutional Review Board at the Women and Children's Hospital at Buffalo (WCHOB). The study was initiated in 2002 and was a retrospective review of paper charts of hospitalized patients who had been diagnosed with reflux prior to 12 months of age and who were 2 years or older at the time of the study. Five years of paper charts, between January 1, 1995 and December 31, 1999 at the WCHOB were reviewed. Charts were selected by ICD-9 codes (Table 1) [4] that would capture discharges coded as reflux. To be included in the data analysis, in addition to an appropriate ICD-9 code, a diagnosis of reflux prior to 12 months of age had to be documented in the chart. Reflux is a clinical diagnosis and while there are inherent problems in relying on clinicians to make a diagnosis, in fact, clinicians drive the studies, interventions and outcomes. We further characterized the patients as having primary reflux, that is, reflux was the primary diagnosis, or secondary reflux, reflux was not the primary diagnosis. Of necessity the group characterized as secondary reflux was not uniform in disease, length of hospital stay or interventions. Finally, we identified infants with reflux born at < 37 weeks gestation.

**Table 1:** ICD-9 Codes

530.81	Esophageal reflux
530.11	Reflux esophagitis
787.03	Vomiting alone
564.03	Vomiting following gastrointestinal surgery
307.5	Eating disorder
779.3	Feeding problems in the newborn
933.1	Choking due to food regurgitation
578.0	Vomiting blood
306.4	Psychogenic vomiting

Using questionnaires from the literature as well as personal experience, a codebook was constructed to transform extracted data to numerical form. The following categories were covered in the codebook: demographics, clinical data, laboratory data and environmental data. However, only demographic data is reported herein.

### Data Collection and Entry

A single individual (CR) reviewed each chart meeting the inclusion criteria. Data were entered into a Microsoft Access (Microsoft, Redmond, WA) database using the codebook. Ten percent of the charts (110) were also reviewed by one of a number of secondary reviewers. The variance in the interpretation of chart entries between the 2 reviewers was 0.1%.

Although special care was taken to identify information associated with fathers, very little data about fathers was contained in the charts. For many variables more than 50% of the charts were missing information. Therefore, information about fathers was excluded from the analysis.

Data were imported from the Microsoft Access database into the SAS (SAS Institute, Cary, NC) software package. Descriptive statistics were generated using frequency tables.

Population data for comparison were obtained from the New York State Department of Health (NYSDOH) [5] United States Census data [6], and the Kaleida Health Information System.

Where possible groups were compared using chi square test.

## Results

There were 98,320 births, 7,155 infants were born at < 37 weeks gestation, in Western New York (WNY) during the study period. For each county, the birth rate per 1,000 females ages 15–44 years as described by the NYSDOH [5] was stable. The largest difference over the 5 year period was 4 births per 1,000 females ages 15–44. The proportion of infants born at < 37 weeks gestation varied with county from a low of 9.7 to a high of 11.7%. The proportion of infants born at < 37 weeks gestation for the state of NY was 11.4%.

**Figure 1:**
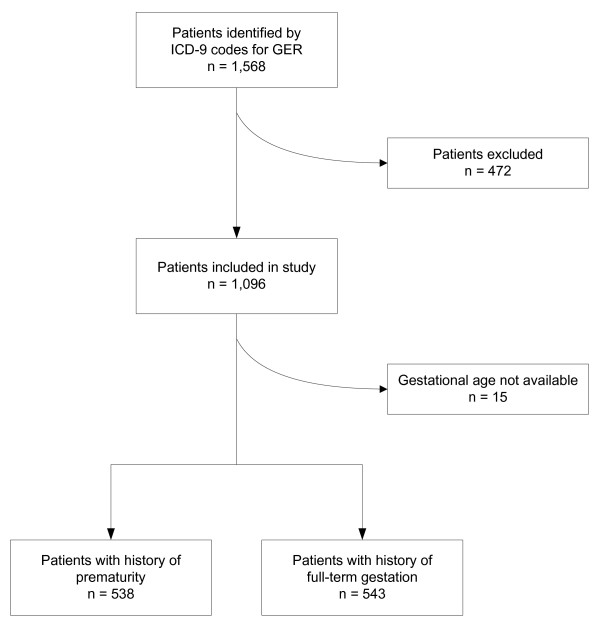
**Discharges with ICD-9 codes consistent with reflux**. There were 1,568 discharges, 472 were excluded because no diagnosis of reflux was made before 12 months of age. A total of 1,096 charts were included in the review. Of the charts reviewed, 514 were from children born ≥ 37 weeks gestation, 519 were from children born at < 37 weeks gestation. No birth data was recorded for 53.

Figure 1 shows the number of patient discharges during the study and the number included and excluded in this study. The number of charts that had missing information was variable and depended on the information sought. For a retrospective review, charts often have missing data, charts may not be available for review, or chart entries may be ambiguous or not extractable so the information available varies with each question.

During the study period there were 16,846 discharges of children ≤ 2 years of age. There were 3,145 discharges of children born at < 37 weeks gestation. This is 3.2% of the total births in the area and 10% of all discharges ≤ 2 years. There were 1,096 children ≤ 2 years of age discharged with the diagnosis of reflux. This is 1.1% of all births and 6.4% of discharges of children ≤ 2 years. Table 2 shows the 10 most frequent discharge diagnoses; reflux was the 7^th ^most common diagnosis.

**Table 2:** Ten most frequent discharge diagnosis 1995–1999

Number	ICD-9	Diagnosis	Number	Percent of discharges^1^
1	466	Bronchiolitis	1282	7.6
2	0799,0479	Viral infections	698	4.1
3	771	Perinatal infections	504	3.0
4	493	Asthma	473	2.8
5	7806	Fever	454	2.7
6	486	Pneumonia	392	2.3
7	53081	Reflux	382	2.3
8	5990	Urinary Tract Infection	273	1.6
9	769	Respiratory Distress	272	1.6
10	00861	Rotavirus infection	260	1.5

^1^number of hospital discharges of children ≤ 2 years (29,957) minus number of normal newborn discharges (13,111) = 16,846

Table 3 shows the fraction of patients for whom reflux was the primary diagnosis, the frequency of hospital discharges, the mean age at diagnosis, sex and birth order. The average length of stay, excluding newborns, was 3.96 ± 0.05 days (range 3.8–4.1 days) during the study period.

**Table 3:** Description of hospitalized children with reflux

	All subjects (N = 1096)^1^	≥ 37 weeks gestation (N = 543)	<37 weeks gestation (N = 538)
Reflux is primary diagnosis	235 (21%)	178 (33%)^2^	53 (10%)
Hospital discharges:			
1	496 (45%)	269 (50%)^3^	223 (41%)
2	228 (21%)	105 (19%)	121 (22%)
3 or more	306 (28%)	148 (27%)	154 (29%)
10 or more	30 (2.7%)	19 (3.5%)	8 (1.5%)
Mean age at diagnosis of reflux (months)	5.9 ± 20.2	6.4 ± 21	4.4 ± 17.1
Male	592 (54%)	275 (51%)	301 (56%)
First-born	504 (46%)	270 (50%)	235 (44%)

^1^Number of patients with missing gestational age = 15.

^2 ^Significant difference between ≥ 37 weeks gestation and < 37 weeks gestation, p < 0.001.

^3 ^Significant difference between ≥ 37 weeks gestation and < 37 weeks gestation, p < 0.01.

About half of the children discharged with a diagnosis of reflux were born at < 37 weeks gestation. Fewer children born prematurely had reflux as a primary diagnosis compared to infants born at term, OR = 0.21 (95% CI: 0.15–0.30). Infants born at < 37 weeks gestation had complicated hospitalizations and reflux was often only one of many problems. Children born at term had fewer complicating medical problems.

Table 4 shows that when reflux was not the primary diagnosis little difference existed between the diagnostic categories for term and preterm infants. The most common reason for hospitalization for all infants, whether born <37 weeks or ≥ 37 weeks was conditions surrounding the perinatal period.

**Table 4:** Frequency of primary diagnoses if not reflux

ICD-9	Description	All Patients	Infants born at ≥ 37 weeks gestation	Infants born at < 37 weeks gestation
0–139	Infections and Parasitic Diseases	25	20	4
240–279	Endocrine, Nutritional, and Metabolic. Immunity	11	11	0
320–389	Nervous System and Sense Organs	10	9	1
390–459	Circulatory System	3	3	0
460–519	Respiratory System	52	38	14
520–579	Digestive System	9	7	2
580–629	Genitourinary System	3	1	2
740–759	Congenital Anomalies	74	54	20
760–779	Conditions in the Perinatal Period	469	77	392
780–799	Symptoms, Signs, and Ill Defined Conditions	90	73	17
800–999	Injury and Poisonings	22	17	5
E codes	Supplementary Classification	10	9	1
V codes	Supplementary Classification	6	5	1
Unknown or missing		62	34	22

Table 5 shows the racial make up of the area, the race of children ≤ 2 years during the study period and the race of children hospitalized with the diagnosis of reflux.

**Table 5:** Race in Western New York and of children hospitalized with reflux during the study period

Group 1995–1999	Population of 8 counties N (% of total)	Hospital admissions ≤ 2 years	Study patients N (%)	Study patients ≥ 37 weeks gestation and reflux N (%)	Study patients < 37 weeks gestation and reflux N (%)
Total population	1,591,726 (100)	29,965 (100)	1096 (100)	514 (100)	519 (100)
White	1,376,950 (86.5)	19,702 (66)	748 (68.3)	381 (74.1)	356 (68.6)
Black	39,437 (2.4)	8,571 (29)	239 (21.8)	107 (20.8)	129 (25)
Other^1^	175,339 (11)	1,498 (5)	109 (10)	26 (5.1)	34 (6.6)

.

^1^Asiatic, American Indian, other, unknown.

Table 6 shows the proportion of mothers who smoke. A higher proportion of mothers of infants who have reflux smoke than the general population of women.

**Table 6:** Parental smoking and reflux

Group	Population^1 ^N (%)	Reflux N (%)	Reflux ≥ 37 w N (%)	Reflux <37 w N (%)
Mother Smokes	1,591,726 (22.2)	385 (35)	182 (34)	198 (37)
Missing	None	83 (7)	54 (9.4)	21 (3.9)

^1^

Table 7 shows the educational level achieved by mothers of children hospitalized for reflux and the educational level achieved by the population in the area. For mothers of infants born ≥ 37 the missing information was too large to allow comment. However, for mothers of infants born at < 37 weeks gestation, a disproportionate number achieved less than a high school education and fewer than the general population had any college education.

**Table 7:** Mother's Education (%)

Group	Population in WNY^1^	All reflux	Reflux ≥ 37 w	Reflux < 37 w
< HS	22.1	18	13.1	22
HS	32.2	17	9.2	25.5
Some College	17.1	7.6	3.1	12.3
Associate	8.8	4.3	0.9	7.6
BA/BS	11.8	4.4	1.7	7.3
Grad	7.9	6.7	0.7	11.7
Missing	NA	45.6	70	20

HS = High School, Associate = associate degree, BA = Bachelor of Science, Grad = studies after obtaining a BA or BS.

^1^.

Table 8 shows that about half as many mothers of infants hospitalized with reflux were employed compared to the women in the work force in WNY. Fewer mothers of infants born ≥ 37 weeks gestation were employed compared to mothers of infants born at < 37 weeks gestation.

**Table 8:** Mothers in labor force (%)

Group	Labor force in WNY, ≥ 16 years of age^1^	Reflux	Reflux ≥ 37 weeks	Reflux < 37 weeks
Women	57	27	23	31
Missing	None	18	25	11

^1^

US census data [6] shows that between 1995 and 1996, 85% of people in the US had some type of insurance, 25% had public insurance, 70% had private insurance and 15% had no insurance. Table 9 shows that during the study period more than 90% of children discharged from the hospital had insurance, 48% had public insurance and 45% had private insurance. Five percent had both and 2% had none.

**Table 9:** Insurance (%)

Insurance	Population^1 ^(%)	Discharges ≤ 2 y	Reflux	Reflux ≥ 37 w	Reflux < 37 w
Any	85	97	93	92	93
Public	24	45	48	49	47
Private	71	52	45	43	47
Both	Not listed	1	5	5	5.4

^1^

## Discussion

This is the first report on the demographics of children with reflux who are sick enough to be hospitalized.

WNY experienced little population flux over the study years and this allowed for an unusually accurate means of extrapolating data. For the purposes of this study WNY includes eight counties: Allegheny, Cattaragus, Chautauqua, Erie, Genesee, Niagara, Orleans, and Wyoming. This is an area bounded on the south by the Pennsylvania state border, on the north by Lake Ontario and on the west by Lake Erie. Because WCHOB serves as the regional center of WNY, and it is the only hospital in the described area that has designated pediatric beds, it is possible to estimate complication rates and rates of medical and surgical therapy for reflux. WCHOB is a 318 bed freestanding pediatric hospital that was established in 1892. It is the pediatric teaching hospital for the State University of New York School of Medicine and Biomedical Sciences and is the only pediatric in-patient facility in WNY. WCHOB is the only facility that offers testing for reflux, including pH probes, scintigraphy, gastrointestinal x-ray, esophageal motility and upper endoscopy. WCHOB is the only major pediatric surgery center in the area that offers laproscopic fundoplication for infants with severe complications of reflux. Therefore, virtually all infants from WNY with severe reflux were evaluated and treated at WCHOB.

For this study we relied on the discharge diagnosis to identify the patients.

Discharge diagnosis that are deemed the reason for hospitalization are recorded. In a systematic review of studies comparing routine discharge statistics the coding of discharge diagnosis accuracy on average was found to be high [7] especially for operations and procedures, less so for routine hospital information.

This study shows that a large proportion of children ≤ 2 years of age in WNY are diagnosed with reflux severe enough to result in hospitalization, or contribute to hospitalization. The NYSDOH estimates discharge rates for asthma, gastroenteritis and otitis media (discharges/10,000/year). During the duration of the study the mean rate of discharges for the 8 county area for children ≤ 2 years was 11.9, 6.7, and 1.7 for asthma, gastroenteritis and otitis media respectively. The discharge rate for reflux was 1.4, less than that for asthma or gastroenteritis, but comparable to otitis media. Both otitis media and reflux are common in children under 2 years of age and are usually minor illnesses. Nevertheless our results show that the diagnosis of reflux has a major impact on rates of hospitalization for children ≤ 2 years of age.

The average length of stay for children with a primary diagnosis of reflux only was longer than the average for the hospital as a whole during this time. Approximately 50% of the study subjects required more than one hospitalization and approximately 30% required three or more. This suggests that children with reflux are frequent users of in patient hospital services.

**Figure 2:**
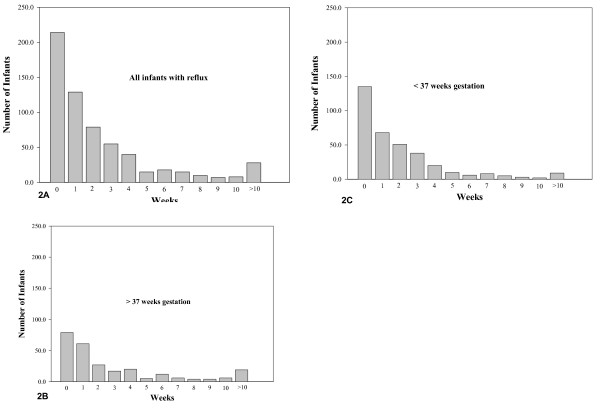
**Figure 2A**, the median time vomiting was present in infants with reflux (N = 960, 88%) was 4.0 weeks before diagnosis. Figure 2B, the median time vomiting was present in term infants with reflux (N = 478, 88%) was 3.0 weeks before diagnosis. Figure 2C, the median time vomiting was present in preterm infants with reflux (N = 473, 88%) was 4.0 weeks before diagnosis. Of the 960 patients, no information on gestational age was available for 9 patients.

Reflux was the primary diagnosis at discharge for 235 (21%) of the patients (Table 3). Four hundred ninety six patients (45%) had 1 discharge during the first 24 months of life, 228 (21%) had 2 discharges and 306 (28%) had 3 or more discharges. Thirty (2.7%) had 10 or more discharges. The age of diagnosis of reflux was 5.9 ± 20.2 months. For those patients who had vomiting (N = 960, 88%) the median time vomiting was present before diagnosis was 4.0 weeks, Figure 2. Sixty eight percent had the symptom of vomiting present for 2 or less weeks at the time of diagnosis. Fifty four percent were male and 46% were first born children. For children with a primary diagnosis of reflux the length of stay was 5.9 ± 0.78 days, range 4.4 to 8.8 days. For children for whom reflux was not the primary diagnosis, the length of stay was 20.3 ± 0.9 days, range 18.1 to 22.6 days

We identified some differences between infants who were born prematurely and those born at term. Of the children discharged with reflux, about half were born prematurely and reflux was the primary diagnosis for about 10% compared to about 20% for those born at term. Children born prematurely often have multiple medical problems and reflux may not be the most severe of those problems. There was no difference between infants born < 37 weeks and ≥ 37 weeks for the number of hospital discharges, mean age at diagnosis of reflux, sex, birth order or length of time vomiting was present before diagnosis.

The population in WNY is predominately Caucasian (86%), 2.4% of the population is African American (AA). Twenty-nine percent of AA children, 3 times the proportion of AA in the population, were discharged from the hospital, but less than 3 times the proportion of AA were discharged from the hospital with a diagnosis of reflux

It appears there is more reflux requiring hospitalization in AA children than in Caucasian children when hospital discharges are compared to the racial make up of the community. However, proportionately, AA discharges far exceed Caucasian discharges. Comparing AA children discharged with a diagnosis of reflux to total AA admissions and Caucasian children discharged with a diagnosis of reflux to total Caucasian discharges, AA are half as likely to be discharged for reflux as Caucasian. This observation is consistent with that of Nazer, et al [8] who showed that based on Ph probe data Caucasian infants have a significantly higher incidence of reflux than AA infants. The disproportionate number of AA discharged from the hospital might be explained by a different frequency of some diseases, different use of medical facilities, socioeconomic factors, or other unidentified factors. Our data does not permit us to discriminate among the possibilities.

Too few of the medical records documented the education level of mothers of infants born at ≥ 37 weeks to comment. But, compared to the area population, a disproportionate number of mothers of infants born at < 37 weeks completed a high school education or had any college education. Luo et al [9] observed increasingly higher rates of preterm birth in mothers with lower levels of education. Our data did not permit us to determine if the lower education of the mothers of infants born at <37 weeks gestation who had reflux was related to reflux or prematurity

In 1997, 23% of the US population and 23% of the New York State population, 25.1% of men and 22.2% of women smoked [10,11]. Despite an aggressive antismoking stance, the proportion of people who smoke in New York state ranged from 22.5 to 22.3 from 1990 through 2000 [12]. Smoking itself is associated with an increased risk of premature births [13]. Passive smoking may be a risk factor for infant reflux. Tobacco smoke induces lower esophageal sphincter relaxation [14] and Alaswad et al [15] showed a strong correlation between esophageal pH and environmental smoke exposure in infants who presented with apparent life-threatening events. However, Martin et al [16] showed no association between environmental smoke and infant spilling. While this data does not permit a robust conclusion about the association of smoking and reflux in children less than 2 years, the disproportionate numbers of mothers who smoke and whose children have reflux is remarkable.

Compared to national insurance data all children discharged from WCHOB were more likely to have medical insurance. However, about 50% of the children in WNY had public insurance compared to about 25% nationally. There was no difference among all discharges of children ≤ 2 years, or those with reflux in WNY. The high proportion of children in this study who had health insurance likely reflects NY State's aggressive program to provide health insurance for children.

## Conclusion

This is the first time that demographic data on children hospitalized with a diagnosis of gastroesophageal reflux has been studied and the data show that reflux is a common discharge diagnosis for hospitalized children ≤ 2 years of age. Frequently reflux is not the primary discharge diagnosis. Children for whom the primary diagnosis is reflux have longer than average hospital stays and about half had multiple hospital admissions during the study period. Those born at < 37 weeks were less likely to have reflux as a primary diagnosis. Mothers of children with reflux are more likely to have less education, to receive public insurance, smoke more, and be unemployed than the average female in the general population in WNY. AA children ≤ 2 years were disproportionately discharged from the hospital compared to Caucasian children. Once admitted to the hospital AA were less likely to have reflux as a diagnosis than Caucasians. This study raises questions about which children with reflux are hospitalized and why and suggests that some demographic factors may make a child more likely to be hospitalized. It is also possible that these factors place the child at risk for severe reflux.

## Competing interests

The author(s) declare that they have no competing interests.

## Authors' contributions

SSB conceived of study, participated in design and coordination of study, drafted manuscript.

CMR performed the chart review and participated in the development of this manuscript. MSL performed the data analysis and participated in the development of the manuscript. RDB conceived of the study, participated in the design and coordination of the study and contributed to the development of the manuscript.

All authors read and approved the final manuscript.

## Pre-publication history

The pre-publication history for this paper can be accessed here:


